# Catarrh

**Published:** 1873-10

**Authors:** 


### CATARRH.

One of our subscribers writes: "In the March number of Hall's Journal of Health you gave us an excellent article upon 'Catarrh and its Cure.' You say, however, that 'if you snuffle anything whatever up the nose, in proportion as it arrests or diminishes the running from the nose, or eyes, or throat, in such proportion it is certain to show itself in the lungs within a very few days, and even in a very few hours, sometimes.' Several physicians of good standing in the profession have recommended the snuffing up the nose of luke-warm water, or cold water with a little salt in it; and one physician in this city says that it will certainly effect a cure if persevered in. We have tried the experiment, and, we thought, with benefit, as it gradually relieved us of the unpleasant feeling in the head and nose; but whether this relief was followed with a cough or not, we cannot, for the life of us, remember."

An estimable lady had a running sore, which failed to be "cured up" by ordinary remedies. One of the neighbors had the reputation of great success in treating such ailments; her services were requested, and the sore did heal in a very short time. In a few days thereafter the patient suddenly died, simply because one of the drains of the system was stopped up. There is only one safe way of arresting any discharges from the human body, especially if of long continuance, and that is to keep the bowels acting freely every day. To stop any such discharge and to allow constipation is always exceedingly hazardous.

Many a person is cured of a cough, the next result being a dangerous if not fatal bleeding from the lungs; hence the importance of always consulting a physician in case of decided illness. To cause one symptom to disappear is not a cure, if, in a short time afterwards, a worse malady manifests itself. A hive on a child may be very troublesome, and it can be driven in; it can be made to disappear, to be followed by convulsions. Persons may try a little warm water or cold salt water in case of a running from the nose; it seems to be simple, but the safer plan is to let it alone, keep the system open, and avoid renewing the cold.

